# Pursuing the Diffraction Limit with Nano-LED Scanning Transmission Optical Microscopy

**DOI:** 10.3390/s21103305

**Published:** 2021-05-11

**Authors:** Sergio Moreno, Joan Canals, Victor Moro, Nil Franch, Anna Vilà, Albert Romano-Rodriguez, Joan Daniel Prades, Daria D. Bezshlyakh, Andreas Waag, Katarzyna Kluczyk-Korch, Matthias Auf der Maur, Aldo Di Carlo, Sigurd Krieger, Silvana Geleff, Angel Diéguez

**Affiliations:** 1 Electronic and Biomedical Engineering Department, University of Barcelona, 08028 Barcelona, Spain; canals@ub.edu (J.C.); vmoro@ub.edu (V.M.); nfranch@ub.edu (N.F.); anna.vila@ub.edu (A.V.); albert.romano@ub.edu (A.R.-R.); dprades@ub.edu (J.D.P.); angel.dieguez@ub.edu (A.D.); 2 Institute for Nanoscience and Nanotechnology-IN2UB, University of Barcelona, 08028 Barcelona, Spain; 3 Institute of Semiconductor Technology, Technische Universität Braunschweig, 38106 Braunschweig, Germany; d.bezshlyakh@tu-bs.de (D.D.B.); a.waag@tu-braunschweig.de (A.W.); 4 Department of Electronic Engineering, University of Rome “Tor Vergara”, 00133 Roma, Italy; katarzyna.kluczyk@uniroma2.it (K.K.-K.); auf.der.maur@ing.uniroma2.it (M.A.d.M.); aldo.dicarlo@uniroma2.it (A.D.C.); 5 Faculty of Physics, University of Warsaw, 00-662 Warsaw, Poland; 6 CNR-ISM, 00128 Rome, Italy; 7 Department of Pathology, Medical University of Vienna, 1210 Wien, Austria; sigurd.krieger@meduniwien.ac.at (S.K.); silvana.geleff@meduniwien.ac.at (S.G.)

**Keywords:** CMOS sensor, nano-LED, optical downscaling, nanopositioners, miniaturization

## Abstract

Recent research into miniaturized illumination sources has prompted the development of alternative microscopy techniques. Although they are still being explored, emerging nano-light-emitting-diode (nano-LED) technologies show promise in approaching the optical resolution limit in a more feasible manner. This work presents the exploration of their capabilities with two different prototypes. In the first version, a resolution of less than 1 µm was shown thanks to a prototype based on an optically downscaled LED using an LED scanning transmission optical microscopy (STOM) technique. This research demonstrates how this technique can be used to improve STOM images by oversampling the acquisition. The second STOM-based microscope was fabricated with a 200 nm GaN LED. This demonstrates the possibilities for the miniaturization of on-chip-based microscopes.

## 1. Introduction

Optical microscopy systems have been diversified over past centuries with a variety of techniques (phase contrast [1,2,3], dark field [4,5], confocal [6,7], fluorescence microscopy [8,9,10], etc.) that have allowed for the easy observation of most processes and cellular structures down to a few tenths of a micrometer. For the exploration of objects on a smaller scale, photonic systems have been hindered by the light diffraction limit, theorized by Ernst Abbe to be approximately 200 nm.

More recently, some super-resolution (SR) techniques, i.e., techniques with a resolving power beyond the diffraction limit, such as stimulated emission depletion (STED) [11,12,13], structured illumination microscopy (SIM) [14,15], stochastic optical reconstruction microscopy (STORM) [14,15], photo-activated localization microscopy (PALM) [16,17,18], or scanning near-field microscopy (SNOM) [19], have demonstrated resolutions of as small as a few tenths of a nanometer. However, the overall complexity and cost of these imaging systems have increased significantly, limiting the widespread use of some of the more advanced optical imaging techniques beyond well-equipped laboratories.

Alternatively, cheaper and simpler techniques are being investigated. For instance, lensless microscopy [20] takes advantage of the large field-of-view (FOV) and small pixel size provided by large commercial cameras (CMOS or CCD). Various methods of lensless imaging can be found today, whether based on shadowing [21,22,23], fluorescence [24,25,26], holography [27,28,29], or 3D imaging [30,31]. The refinement of high-resolution (HR) techniques and the improvement of computational resources [32,33,34] have boosted the spatial resolution below the pixel size limitation. Thanks to techniques such as pixel super-resolution (PSR), based on the displacement of the image sensor [35,36,37], the sample or the light source, they can reach resolutions of lower than 1 µm [38]. However, the diffraction limit remains a barrier for low-cost SR alternatives.

**Figure 1:**
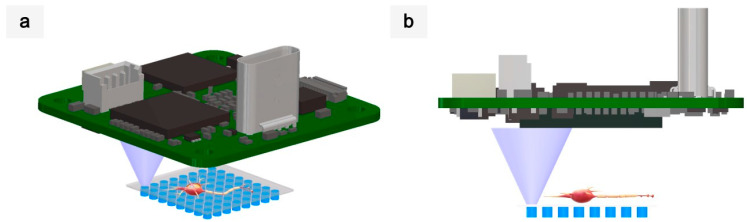
Schematic diagram of electronic scanning transmission optical microscopy. (**a**) Perspective view. (**b**) Side view.

A novel microscopy imaging technique has emerged, which harnesses the key features of lensless microscopy and scanning techniques. As in lensless microscopy, LEDs are the basic elements. However, instead of fixing its position, the LED is moved to scan the sample, as in scanning microscopes. This scan can be performed with one LED alone, by moving it over the sample or by moving the sample over the LED; alternatively, its apparent position can be electronically controlled using the LEDs available in an array or in an LED micro-display (see Figure 1). The other key component of the microscope is an optical photodetector used to measure the light traversing the sample, and a camera can typically be used. The first prototype using this technique was already presented in [39], where every LED in an 8 × 8 gallium nitride (GaN) array (5 µm in size and 10 µm in pitch) was sequentially turned on and off, mapping the sample to prove the principle of operation of nano-illumination microscopy (NIM). The main features of this technique are that the resolution is given by the LED pitch and that the FOV is given by the LED array size when the sample is placed in direct contact with the array. Therefore, the sensing device used does not play as important a role as in conventional lensless microscopy. NIM only needs two chips and benefits from being relatively close together. On the other hand, LED miniaturization is a very active field of research, which seems to allow for sizes smaller than Abbe’s diffraction limit [40,41,42]. In this context, the fabrication of new compact low-cost microscopes based on nano-LED scanning transmission optical microscopy (STOM) is pursued, capable of sub-micron resolutions. With the decrease in LED size, further resolutions can be achieved [43]. Nevertheless, high-resolution and large-FOV microscopes remain a still-distant prospect because of the research required, which is limited to advancements in both directions.

In this work, the second and third generations of NIM microscopes are presented. Both are based on [39], where the proof-of-concept and the characterization of the imaging technique were described. The first microscope presented was assembled with a single 5 µm LED, which was optically downscaled with a lens to further investigate the possibilities of the STOM technique. As this approach integrated an optical lens, the resulting spot was diffracted, creating a virtual LED with a size determined by the first minimum of the light intensity from the optical axis, i.e., the size of the diffracted spot is given by

(1) $$D_{\mathrm{spot}}=\frac{1.22\cdot \lambda }{\mathrm{NA}},$$

where λ is the wavelength of the LED-emitted light and NA is the numerical aperture of the lens.

Nevertheless, as the distance between LEDs, or their position, is also downscaled, the light can be shifted onto the sample with steps that are smaller than the diffraction limit, allowing for an oversampling of the image. Contrary to [39], where the sample is scanned by the light sequentially emitted by the 8 × 8 nano-LED array, a single LED and a set of nanopositioners are used in order to carry it out. Therefore, the nanopositioners are integrated to move the sample, i.e., they emulate the light scan, with nanometric precision. While the demagnified version of the 5 µm LEDs is used for the study of the STOM microscope with optical downscaling, the limits of the technique by building a second STOM microscope are explored, based on the 200 nm LEDs below the diffraction limit for the wavelength emitted. The fabrication and characterization of the 200 nm GaN-based nano-LED array used were reported in [44].

To demonstrate the potential of the methods, a number of metal nanostructures with different sizes and shapes were fabricated by electron-beam lithography (EBL) and were observed with the two microscopes. Additional experiments were performed with the aim of potential application in the biology field. Therefore, the resolution limit has been pursued with different versions of STOM.

In the following sections, the Materials and Methods are detailed, followed by the Results and Discussion sections. This includes the description of the STOM prototype, using an optically downscaling LED array, and the experimental results obtained with it, to delve into the role of the scanning step. In the second section, the STOM prototype is described using 200 nm nano-LEDs, together with the experimental results. Finally, the main conclusions are summarized.

## 2. Materials and Methods

### 2.1. CMOS Sensor

The board camera used (TIS-DMM-22BUC03-ML) integrates an Aptina MT9V024 CMOS sensor (from OnSemiconductor^®^, Phoenix, AZ, USA) with a sensing area of 4.55 mm (H) × 2.97 mm (V). The sensor has a frame rate of 76 fps @ 744 H × 480 V resolution (higher frame rates at lower resolutions), a 6 µm × 6 µm global shutter pixel with a quantum efficiency of ~50% in the blue range (450–495 nm), and an 8-bit dynamic range. The board camera includes a selectable 10- to 8-bit ADC and a bit-serial LVDS or parallel data interface. In addition, it also supports an 8-bit monochrome pixel format. Therefore, this format transmits data using one byte for each pixel. The Python interface controls the sensor and the electromechanical setup to perform the image acquisition.

### 2.2. Nanopositioning System

The fine nanopositioning system is formed by a compact XY piezo stage and a vertical Z piezo stage from Physik Instrumente (PI). P-621.2CD and P-621.2ZCD nanopositioners (from PhysikInstrumente GmbH & Co. KG, Karlsruhe, Germany) are used on the XY plane and in the Z vertical direction, respectively. In the closed-loop configuration, both actuators had 0.4 and 0.3 nm resolutions. As for the travel range, i.e., the maximum FOV, this reaches up to 100 µm. In addition, a linearity error of 0.02% and a repeatability error of 1 nm are taken into account when performing the scans. The actuator step unit defines the pitch of the emulated LED array. For every complete scan in the XY direction, a frame is captured and processed. The Z-axis actuator is used to place the sample in direct contact with the surface of the LED chip. The coarse system is composed of three N-470 PiezoMike linear actuators coupled to triaxial mechanical stages. Its resolution reaches 20 nm and its travel range reaches 7 mm. However, this system lacks an encoder, so the accuracy of the coarse-motor steps is limited.

### 2.3. Image Reconstruction

In STOM-based microscopes with LED scanning, the sample is placed between the optical detector and the nano-LED array. Then, the sample is mapped by the LED, moving the sample sequentially along the X and Y axes to emulate a nano-LED array. The CMOS detector senses the light transmitted (or blocked) by the object. The image is reconstructed following the same order as the sample movement sequence. The morphology of the sample affects the light received by the sensor. These variations, in terms of light intensity, allow for the reconstruction of the image of the sample in grayscale, according to the sample’s physical properties.

### 2.4. Simulations

The simulations were performed by using the finite-difference time-domain method implemented in the commercial software, CST Studio. All of the calculations were made for wavelengths equal to 450 nm. The optical properties of the materials were modelled with the experimental data available in the literature accounting for real and imaginary parts of the dielectric function. The values used in the calculations were as follows: GaN [45] (ε’ = 5.825, ε’’ = 0.407), Au [46] (ε’ = −1.756, ε’’ = 5.299), SiO_2_ [47] (ε’ = 2.165, ε’’ = 0.007). The emission multiple-quantum-well (MQW) layer was approximated with a dipole source placed in the middle of the MQW layer (i.e., 300 nm underneath the top surface of the LED) and below the center of the Ti/Au contact. The calculations were performed for the x and y polarizations of the dipole source and were then summed up to simulate the unpolarized characteristics of the light emission from the LED. To avoid reflection from the borders of the computational area, open boundary conditions were used in all directions.

### 2.5. Observed Samples

The fabrication of the EBL sample included a device-quality starting material of 4” fused silica wafers, which were 0.525 mm thick. First, dehydration was carried out in an oven at 250 °C for 2 h. Then, a nominally 180 nm-thick CSAR-P6200.09 positive photoresist (from Allresist GmbH, Strausberg, Germany) was spun at a speed of 4000 rpm for 1 min and was cured at 180 °C for 3 min. Prior to resist exposure, a 20 nm-thick aluminum layer was thermally evaporated at 0.3 nm/s to reduce charge build-up in the wafers, while the exposure was performed at a beam current of 2 nA. The sacrificial aluminum layer was removed by a 60′′ single puddle in a 2.38% tetramethyl-ammonium hydroxide solution, and development of the CSAR photoresist was achieved in the AR 600-546 developer (from Allresist GmbH, Strausberg, Germany) for 1 min. The sample was further covered by a 40 nm-thick electron-beam-evaporated chromium layer, deposited at 0.5 nm/s. The lift-off of the deposited metal was performed in Remover 1165 at 45 °C for 20 min, followed by rinsing in isopropyl alcohol.

Human lung fibroblast LL47 (Mado, ECACC, #90102538, Salisbury, UK) were cultured under standard cell culture conditions following the manufacturer’s protocols. Samples for microscopy were prepared in short by tryptic release. After washing with minimum essential medium (MEM, Gibco, 11095080), containing 10% fetal calf serum and 1% penicillin-streptomycin (Gibco, 10378016), cells were counted and 3000 cells were cultured overnight in droplets of 300 µL on 24 × 50 mm coverslips (VWR International, #631-0148) coated with 5 µg/mL of bovine collagen-I (Gibco, A1064401). On the next day, cells were fixed for 20 min at room temperature with MEM containing 2% paraformaldehyde, they were washed twice with phosphate-buffered saline, and they were stained with hematoxylin and eosin, following standard procedures. Finally, cells were embedded with Fluoromount-G (Invitrogen, #00-4958-02) using 22 × 22 mm coverslips (Corning, #CORN2850-22). Cell specimens were assessed using a microscope using a 488 nm LED laser at 2% transmission and an EC Plan Neofluar 10×/0.30 lens (Zeiss). Images with a size of 1612 × 1612 pixels (1.28 × 1.28 mm) and a spatial resolution of 0.79 × 0.79 µm were produced using the transmission photo multiplier of the microscope.

## 3. Results and Discussion

### 3.1. Scanning Transmission Optical Microscope with an Optically Downscaled LED

**Figure 2:**
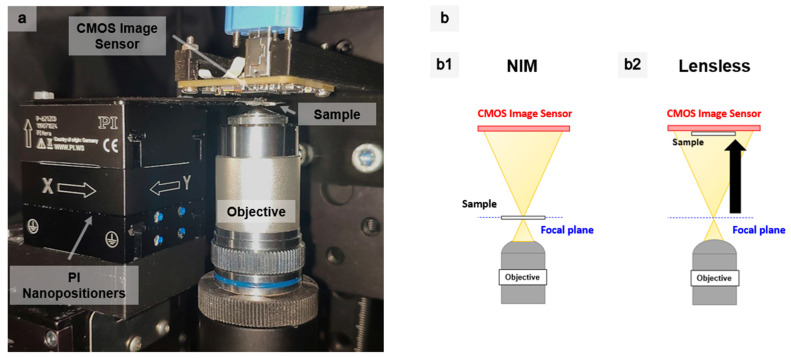
(**a**) Setup of the scanning transmission optical microscope with optical downscaling. (**b**) Schematic diagrams of the STOM with optical downscaling in different operating modes. (**b1**) NIM mode. (**b2**) Lensless mode.

In order to explore how scanning is affected by shifting the light source in steps smaller than the diffraction limit, the first prototype with a lens to provide demagnification was constructed. To do this, the sample was scanned with the downscaled image of a 5 µm LED from the 8 × 8 GaN array described formerly in [39]. Optical downscaling was performed with a x60 objective with an NA of 0.85. Consequently, a reduced and diffracted image of the LED was obtained, ~750 nm in size, according to Equation (1). The Materials and Methods section describes the fine positioning system formed by two piezo stages from Physik Instrumente (PI), with the coarse system used to extend the FOV up to several millimeters and the CMOS camera used for light sensing. The core of this setup is presented in Figure 2a.

A key improvement of this setup, following the results of [39], was the ability to locate the sample precisely where the spot was of minimum size, by placing it at the focal plane of the lens (see Figure 2b1). As the spot size, as well as the pitch, is critical for image formation, this allows for improved resolutions. Additionally, samples that present an inhomogeneous surface can be explored by scanning them at different height levels. Therefore, more information is compiled from the scanned sample due to the in-focus and out-of-focus areas. An additional strength of this microscope was the flexibility in finding the region-of-interest (ROI). Thanks to the large travel range of the coarse piezo-actuators, the sample could be brought closer to the optical detector, thus obtaining an image with a larger FOV, only limited by the detector size, as in conventional lensless microscopy (see Figure 2b2). This allowed for the easier scrolling through the sample.

**Figure 3:**
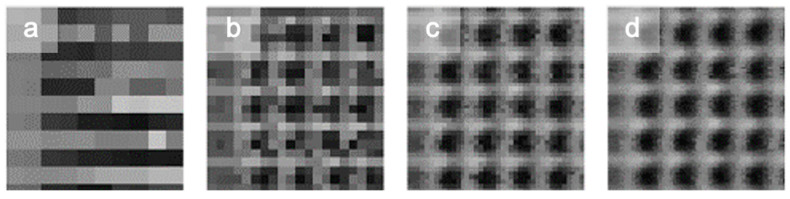
STOM images of an 800 nm-wide squared pattern using a diffracted spot of 750 nm with an objective of x60, with steps of (**a**) 750, (**b**) 400, (**c**) 200, and (**d**) 100 nm.

**Figure 4:**
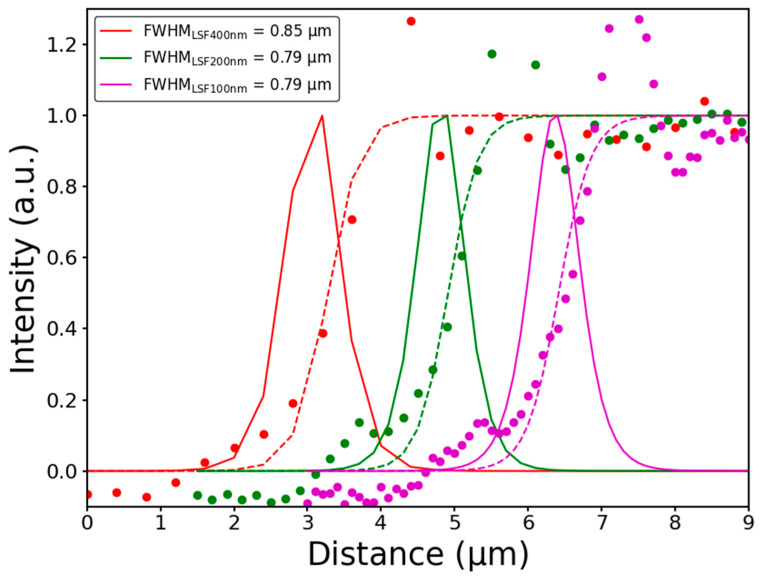
The measured ESF from a sharpened line pattern with steps of 400, 200, and 100 nm. The LSF has been fitted and illustrated with a solid line and has been normalized to unity. The peaks at the top of the sample profile correspond to the diffraction of the object. The raw curves correspond to the same sharp profile, but for easy visual understanding, they have been divided along the X-axis. The resolution calculated from the LSF is shown in the inset.

The effect of the step size on the image reconstruction with this setup can be observed in Figure 3 and Figure 4. Figure 3 presents several images of an 800 nm square pattern array taken in steps of 750 nm (Figure 3a), 400 nm (Figure 3b), 200 nm (Figure 3c), and 100 nm (Figure 3d) with the objective of x60 with an NA of 0.85. Figure 3a shows that, when the step used was near the size of the diffracted spot, the structures could be estimated but not resolved. The starting position of the scan influenced the reconstruction of the image, as it determines whether light intensity is captured just above each square or in the separation between the two. In this case, the size of the LED was equal to the size of the pitch used. However, for a pitch smaller than or equal to the size of the virtual LED, the images were resolved with more clarity (Figure 3b–d), i.e., the measurement was oversampled, which allowed us to improve the acquisition of the STOM image.

Figure 4 shows the corresponding edge spread function (ESF) and line spread function (LSF) [48]. The ESF is the contrast profile across an edge in the image, and the LSF is its derivative. In the images obtained, both were calculated at one edge of a square in the pattern in Figure 3. The resolution can be calculated from both ESF and LSF. This corresponds to the distance between 10% and 90% of the ESF. On the other hand, this can also be evaluated as the FWHM of the LSF. The ESF data were normalized and averaged (dashed lines) to obtain a uniform LSF (solid lines). As can be observed from the data in Figure 4, the resolution was not affected by the step size, although oversampling produced visually improved images. For all step sizes, the resolution of all of them was maintained at around 800 nm, i.e., around the spot size.

**Figure 5:**
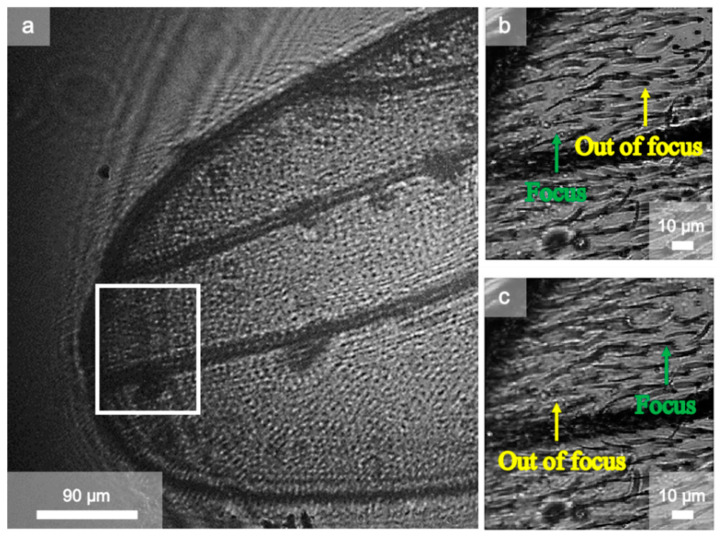
(**a**) Raw image in lensless mode of the fly wing sample. The refocusing images for the selected region of (**b**,**c**) corresponding to the height difference of 2 µm at 17 and 19 µm, respectively.

The final experiments demonstrated the capabilities of the technique for biological microscopy. Figure 5a shows the raw view of a scanned fly wing in the camera. Figure 5b,c show STOM images with the objective of x60 with an NA of 0.85 and a step size of 300 nm. As shown in Figure 5b, regions of the sample were out of focus while others were perfectly focused. Nevertheless, as the focus could be controlled by placing the sample at different heights, it could be refocused, as shown in Figure 5c.

**Figure 6:**
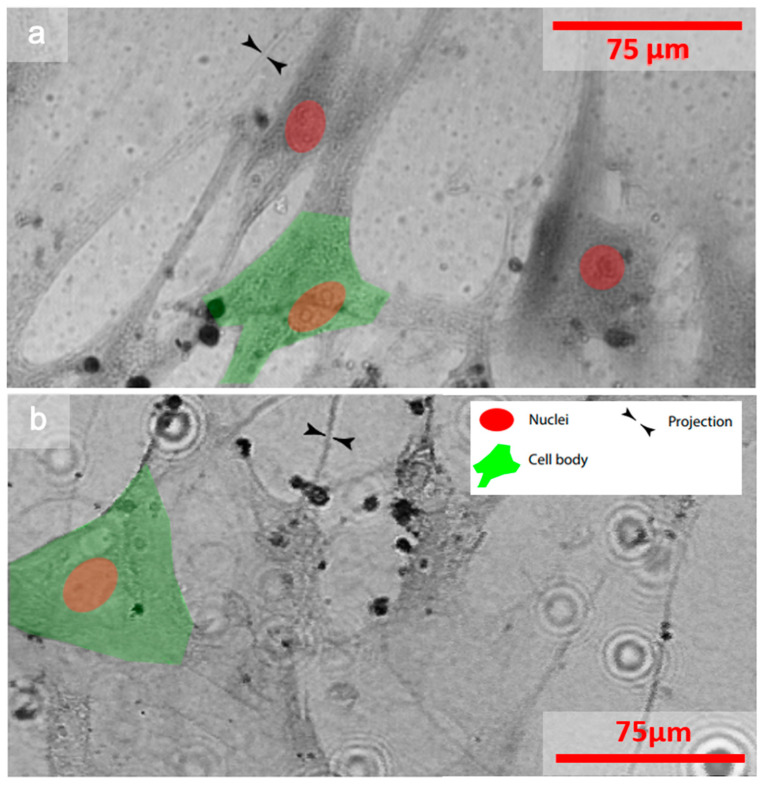
Human lung fibroblasts observed with a (**a**) confocal laser scanning microscope (CLSM) and (**b**) STOM microscope using a diffracted spot of 750 nm and a step size of 600 nm.

In addition, a second sample, composed of a human lung fibroblast, was explored. Figure 6a shows the raw image, recorded with a Zeiss Confocal Laser Scanning Microscope (CLSM) with a pixel resolution of 0.79 µm, which is similar to that of our optical demagnification STOM microscope. A STOM image, with the objective of x60, an NA of 0.85, and a step size of 600 nm of the same set of cells, is presented in Figure 6b. Both the nuclei and the cell bodies, as well as the sample projections, were very similar in size and structure in both images. Therefore, it was possible to analyze the state of the cells thanks to the fact that the nuclei, i.e., the center of mass of the cell, could be identified despite the lower contrast of the STOM image. The two images do not correspond exactly to the same region because, once the sample is scanned, it is dried. Therefore, the images were obtained with different sets of cells that were part of the same cell culture.

### 3.2. Scanning Transmission Optical Microscope with a 200 nm LED

**Figure 7:**
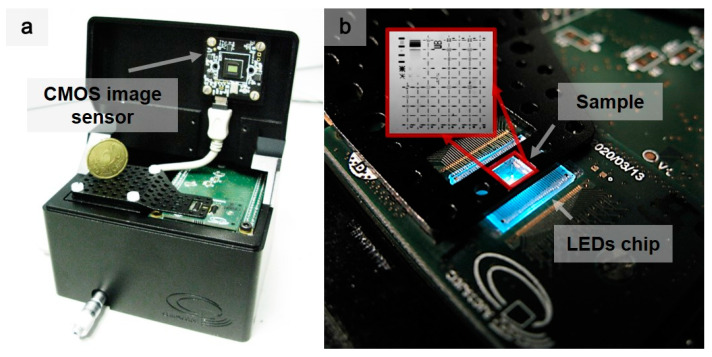
(**a**) Prototype of the mechanical-based microscope in comparison with a 20-cent coin. (**b**) Placement of the sample on the LED array for scanning with the mechanical-based STOM microscope. The operating LED had a size of 200 nm and emitted at a wavelength of 465 nm.

The setup of the microscope used in the second STOM prototype is presented in Figure 7a. The nano-LED light source was obtained from a single GaN chip with an array of 32 × 2200 nm LEDs [44], specifically targeting this new approach to optical microscopy. One of these 32 × 2 LEDs was chosen for the experiments. The scan operation was performed by the displacement of the sample over the LED with a nano-positioning system in raster scan mode, emulating a virtual LED array of arbitrary size with only one LED. When the cover was closed, the camera and the LED chip had an observation chamber of ~1.8 mm.

Figure 7b presents the sample positioning in closer detail. The sample holder was designed with two main openings. The small one was used to fit the sample. The large one was used as an opening for the LED chip bonding. This solution allowed for directly placing the sample on the LED chip to perform the scan in order to obtain the maximum resolution. Two additional micrometer heads were integrated in the microscope base to make a coarse exploration of the sample. Finally, the LED current driver and the control electronics were placed under the LED chip. The current driver allowed for the control of the current of the nano-LED to provide the optimal operation conditions.

**Figure 8:**
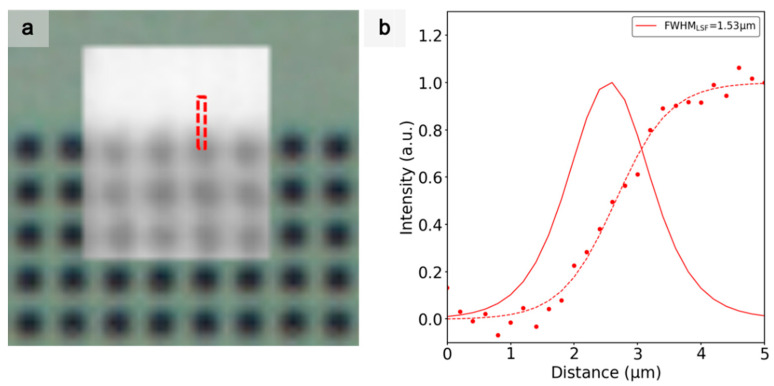
(**a**) STOM image obtained with an LED size of 200 nm biased at 800 nA and with a pitch of 200 nm, superimposed on the standard optical microscopy image at x60 of an EBL matrix of 1.6 µm squares. (**b**) ESF and LSF from a sharpened line with steps of 200 nm calculated in the region marked in (**a**). Both functions were fitted and normalized to unity, and they are illustrated with dashed and solid lines, respectively.

Figure 8a shows the image obtained using an optical microscope (background) with a x60 magnification from the objective, together with the image obtained by the lensless STOM, scanning a region of 80 × 65 pixels using the 200 nm LED (front) of an electron-beam lithography (EBL) sample with 1.6 µm-width squared features. It can be observed that the squares were roughly resolved in the scanning microscope.

As in the previous section, the ESF and the LSF were considered to determine the spatial resolution. Therefore, the ESF and the LSF of the region marked in Figure 8a are presented on Figure 8b. The best obtained resolution was 1.53 µm, close to the actual spacing between squares in Figure 8a, but this was noticeably larger than the LEDs’ dimension and pitch.

**Figure 9:**
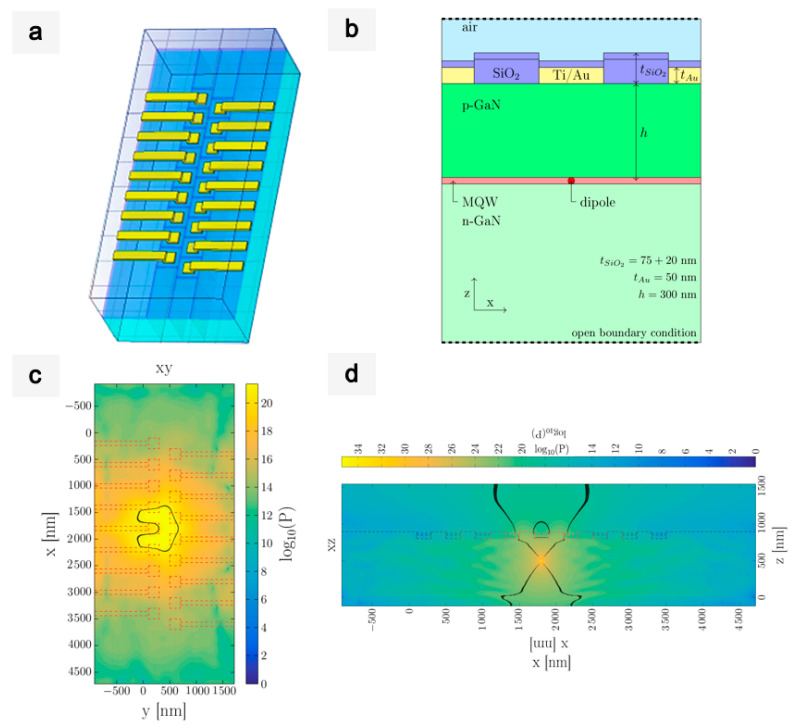
Scheme of the LED model. (**a**) Perspective. (**b**) Cross section along an LED line (scale is not respected). The dipole source was placed at h_1_ = 300 nm under the GaN surface. (**c**) Logarithm of absolute value of power intensity distribution on the XY plane. The cross section was taken at z = 975 nm (i.e., 100 nm above the top surface of the LED). The black lines indicate the field values equal to half of the maximum of the signal. (**d**) Logarithm of absolute value of power intensity distribution in the XZ plane. The cross section was taken at x = 1800 nm.

To evaluate this resolution discrepancy, we present below a theorical analysis of LED behavior. A model based on a reduced version of the 200 nm LED chip was created. Figure 9a presents the scheme of this proposed model, consisting of two lines of nine LED pixels (with a pixel width of 200 nm and pitch of 400 nm) in the line direction. In Figure 9b, the schematic cross-section of the LED model is shown. The system was simulated using the finite difference time domain method, employing the commercial software, CST Studio (see the Materials and Method section). The model includes the Pd/Ti/Au contact on top of each LED. The emitted optical power, predicted by the simulations, is presented in Figure 9c. The results show that there was a local minimum of light intensity above the Au contact, which was due to parasitic absorption by the metal. The shape of the light spot near the top surface of the LED was ring-like around the pad, and the light was emitted mostly between contacts. The same simulation was represented on the XZ plane (Figure 9d). The dipole was placed 500 nm below the LED chip surface. The propagation of light was calculated between the black lines across the Z axis. The width of this area defines the spot size, i.e., the FWHM. Thus, the measured spot size was significantly larger than that of the pixel, due to the influence of parasitic absorption of the metal contacts on light propagation.

**Figure 10:**
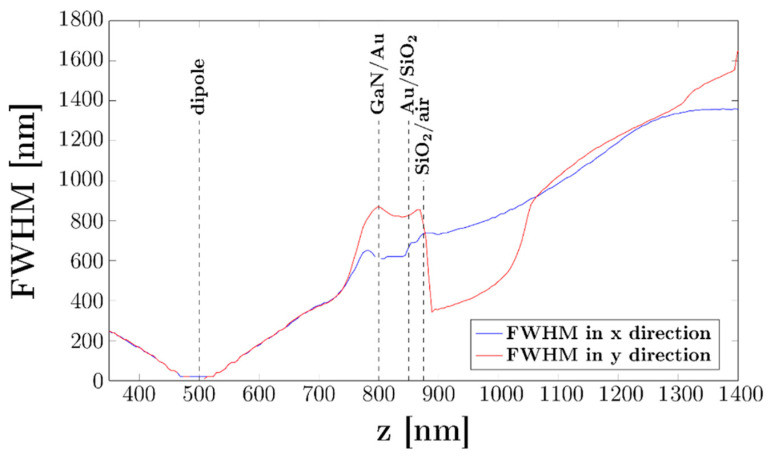
Full-width at half-maximum as a function of the vertical coordinate z. Dashed lines indicate the material interfaces inside the LED structure. The p-GaN layer’s thickness is equal to 300 nm.

Finally, the previous increase in the FWHM in the X and Y axes as a function of distance in the Z-axis is presented more clearly in Figure 10. The LED surface (SiO_2_/air) was 400 nm above the emission region, giving a minimum light spot size of ~800 nm with the sample laying completely flat on the surface. Therefore, according to the simulations, the sample was placed between 400 and 500 nm with respect to the LED surface. Although the maximum achievable resolution would theoretically be 800 nm, this was because the sample could not be moved closer to the emission zone. This limitation can be explained either because the distance of the metal pattern from the LED structure was roughly 500 nm or because the effective emitting area was larger than that assumed for the simulations as a result of lateral carrier diffusion.

Compared to conventional confocal and lensless microscopy, some similarities and differences are evident. In the case of lensless microscopy, a minimum distance to the LED is needed for the sample to be fully illuminated. In confocal microscopes, an optical system is required to focus the sample. However, the STOM technique makes it possible to reduce the distance between the sensor and the light source. This is highly advantageous, as it allows for the manufacturing of smaller high-resolution devices, as the total height of the microscope is equivalent to the thickness needed to introduce the sample. Nevertheless, the current state of STOM technology requires nanopositioners that increase its cost and volume. One solution is to replace the scanning system, based on a single LED, and the mechanical system with LED arrays. These systems currently have pitches similar to image sensor pixels but still have the potential for further reduction as LEDs are continuously being miniaturized. Finally, and despite having a prototype that incorporates LEDs with sizes below the diffraction limit, the behavior of the LEDs was not as expected, and further development needs to be realized to compare their resolution with super-resolution microscopes.

## 4. Conclusions

In this work, we investigated different aspects of scanning transmission optical microscopes based on nano-LED light sources. The method of using a single LED for scanning the sample was validated, as with the prototype based on optical demagnification and the 200 nm LED-based microscope.

The former presents some advantages compared with the previous lensless setup. The possibility of exploring a sample, both inorganic and organic, placed at the lens focus, is interesting because it allows for more than just flat samples to be investigated. Compared with the CLSM system, the use of a less energetic LED source can avoid cell damage in the study of living tissue [49]. Resolutions below 1 µm were achieved using the appropriate optics. Image processing was not required in any of the performed experiments. Scanning the sample with a movement step below the pixel size gave rise to oversampling and improved image reconstruction. However, the use of nanopositioners was a serious drawback, as it limited the prototype in terms of cost. As noted earlier, this can be solved by using LED arrays with large dimensions instead of a single LED.

On the other hand, the second prototype was fabricated with 200 nm nanoLEDs, i.e., with a size below the diffraction limit. At current, our LED manufacturing procedure provided us with an effective resolution of around 1.6 µm. The proper operation of the STOM microscopes without optical elements requires the sample to be in direct contact with the surface of the point of emission of each LED; however, with the passivation and structures present in current technology, this is not possible, which negatively affects the resolving power. 

The resolution of the STOM prototype was worse than expected. Further experiments integrating arrays of nano-LEDs fabricated with a different structure, i.e., transparent contacts (TCO) [50], will prove promising in terms of spot shape and FWHM. It is expected that a more symmetric dot shape and a narrower FWHM can be obtained, thus improving the resolution. Moreover, in combination with the rapid development of new display technologies [51,52,53], i.e., large electronically controlled arrays of LEDs, we could create new prototypes with resolutions close to the diffraction limit with low computational effort and relatively low dimensions and cost.

## Data Availability

Data sharing not applicable.
